# Influence of Polymer Solvents on the Properties of Halloysite-Modified Polyethersulfone Membranes Prepared by Wet Phase Inversion

**DOI:** 10.3390/molecules26092768

**Published:** 2021-05-08

**Authors:** Amanda Grylewicz, Kacper Szymański, Dominika Darowna, Sylwia Mozia

**Affiliations:** Department of Inorganic Chemical Technology and Environment Engineering, Faculty of Chemical Technology and Engineering, West Pomeranian University of Technology in Szczecin, ul. Pułaskiego 10, 70-322 Szczecin, Poland; amanda.grylewicz@zut.edu.pl (A.G.); kacper.szymanski@zut.edu.pl (K.S.); dominika.darowna@zut.edu.pl (D.D.)

**Keywords:** membrane, halloysite nanotubes, *N*,*N*-dimethylformamide, *N*,*N*-dimethylacetamide, 1-methyl-2-pyrrolidinone

## Abstract

Ultrafiltration polyethersulfone (PES) membranes were prepared by wet phase inversion. Commercial halloysite nanotubes (HNTs) in the quantities of 0.5 wt% vs. PES (15 wt%) were introduced into the casting solution containing the polymer and different solvents: *N,N*-dimethylformamide (DMF), *N*,*N*-dimethylacetamide (DMA), or 1-methyl-2-pyrrolidinone (NMP). The type of solvent influenced the membranes’ morphology and topography, as well as permeability, separation characteristics, and antifouling and antibacterial properties. The membranes prepared using DMA exhibited the loosest cross-section structure with the thinnest skin and the roughest surface, while the densest and smoothest were the DMF-based membranes. The advanced contact angles were visibly lower in the case of the membranes prepared using DMF compared to the other solvents. The highest water permeability was observed for the DMA-based membranes, however, the most significant effect of the modification with HNTs was found for the NMP-based series. Regardless of the solvent, the introduction of HNTs resulted in an improvement of the separation properties of membranes. A noticeable enhancement of antifouling performance upon application of HNTs was found only in the case of DMF-based membranes. The study of the antibacterial properties showed that the increase in surface roughness had a positive effect on the inhibition of *E. coli* growth.

## 1. Introduction

The growing demand for clean water and the increasing pollution of the environment by industry have created a need for the development of modern water/wastewater treatment technologies. Membrane processes, especially pressure-driven techniques, are one of the solutions which could be successfully applied instead of conventional methods. However, they suffer from an undesired phenomenon of membrane fouling, which causes the decline of process efficiency over time. There are several approaches for fouling mitigation. One of the most promising is the modification of membranes with nanoparticles (NPs), such as TiO_2_, SiO_2_, Al_2_O_3_, halloysite nanotubes (HNTs), carbon nanotubes (CNTs), titanate nanotubes (TNTs), and others [1]. Among them, HNTs have attracted significant attention as a natural, cheap, and non-toxic material [2,3,4,5,6,7,8]. The application of these NPs was reported as an efficient approach to increase membranes’ permeability, as well as mechanical and thermal resistance [9]. The addition of HNTs was also found to improve the antifouling performance of polymeric membranes during filtration of organic foulants due to an increase of membranes’ hydrophilicity [10]. However, reports in the literature on the effect of the neat HNTs on the antibiofouling properties of the mixed-matrix membranes are very scarce. In the case of biofouling, the main factor contributing to the deterioration of the membrane performance is the biofilm formed due to bacterial growth. The neat HNTs were reported to not exhibit any significant antibacterial performance when added to poly(lactic acid) (PLA) packaging films at 5 wt% [11]. Therefore, modification of HNTs with some biocidal agents such as silver, copper, and *N*-halamine is usually proposed [9,12,13,14,15,16,17].

One of the most common polymers applied for the fabrication of the mixed-matrix membranes modified with HNTs is polyethersulfone (PES) [8,18]. The membranes are usually prepared via the wet phase inversion method (called also the non-solvent induced phase separation process, NIPS). In this technique, a solution of polymer in solvent is cast as a thin film or extruded as a capillary, and subsequently immersed in a non-solvent (coagulation) bath. Typically, water is used as the non-solvent. After immersion of the polymer film in the coagulation bath, the phase separation occurs due to the solvent–non-solvent exchange. The structure of the formed membrane depends on various parameters related to the composition of the casting dope and coagulation bath, as well as casting conditions. Moreover, the morphology and properties of the membrane can be changed by the application of various post-treatment procedures, e.g., drying under room or elevated temperature or freeze-drying [19,20,21]. Amongst these parameters, the polymer solvent plays an important role [22]. Depending on the solvent, various membrane morphologies can be obtained (loose/dense skin layer, a sublayer of various porosity, different shapes of macrovoids, finger-like/sponge-like structure, etc.) [23,24,25].

The most appropriate PES solvents are *N,N*-dimethylformamide (DMF), *N,N*-dimethylacetamide (DMA), 1-methyl-2-pyrrolidinone (NMP), and dimethyl sulfoxide (DMSO) [18,26,27,28,29], although recently a possibility for the application of “green solvents” has also been investigated [30]. The selection of a proper polymer solvent is based on the solvent–polymer–non-solvent interactions. A useful parameter explaining the influence of these interactions on membrane fabrication is the Hansen solubility parameter [31,32]. The value of this parameter for the PES–solvent interaction is higher for DMF (4.20 MPa^0.5^) and DMSO (5.81 MPa^0.5^) compared to NMP (2.97 MPa^0.5^) and DMA (3.05 MPa^0.5^) [32]. A higher polymer–solvent affinity causes a delay in demixing, and thus a delay in the phase separation process. A delay in demixing was attributed also to the higher viscosity of the DMF- and DMSO-based casting dopes. The membranes obtained under such conditions exhibited lower water permeability, lower porosity, and smaller pore size compared to those fabricated with the application of NMP and DMA [33,34,35]. It was also reported that PES membranes obtained using DMA exhibited a more porous structure and higher permeability compared to those fabricated using NMP [32,36]. Another factor affecting the membrane preparation is the volatility of the polymer solvent [32,37].

The studies on the influence of the polymer solvent on the properties of the mixed-matrix PES membranes modified with NPs are very limited. Ghandashtani et al. [38] investigated the effect of three solvents, NMP, DMF, and DMSO, on the performance of SiO_2_-modified PES mixed matrix membranes. The highest permeate flux and oil rejection were observed in the case of the membrane fabricated using DMSO, due to the largest pore size and the most hydrophilic surface [38]. However, these results are in disagreement with the previously cited papers [32,34,35] on the influence of various solvents on the properties of neat PES membranes. This could be related to different membranes’ preparation conditions and the presence of SiO_2_ in the membrane matrix.

The above-presented overview reveals that the subject literature is lacking in the research on the influence of polymer solvent on the properties of the mixed-matrix PES membranes modified with HNTs. The present work covers this gap. The membranes were obtained via the wet phase inversion method using DMF, DMA, or NMP as PES solvents. The physicochemical properties of the membranes were examined based on scanning electron microscopy (SEM), atomic force microscopy (AFM), and contact angle (CA) measurements. Moreover, the pure water flux and separation characteristics were determined. Furthermore, membrane fouling was investigated using bovine serum albumin (BSA) as a model foulant, while the antibiofouling properties were assessed on a basis of inhibition of bacterial growth using *Escherichia coli* as a model microorganism.

## 2. Results and Discussion

### 2.1. Physicochemical Properties of Membranes

**Figure 1:**
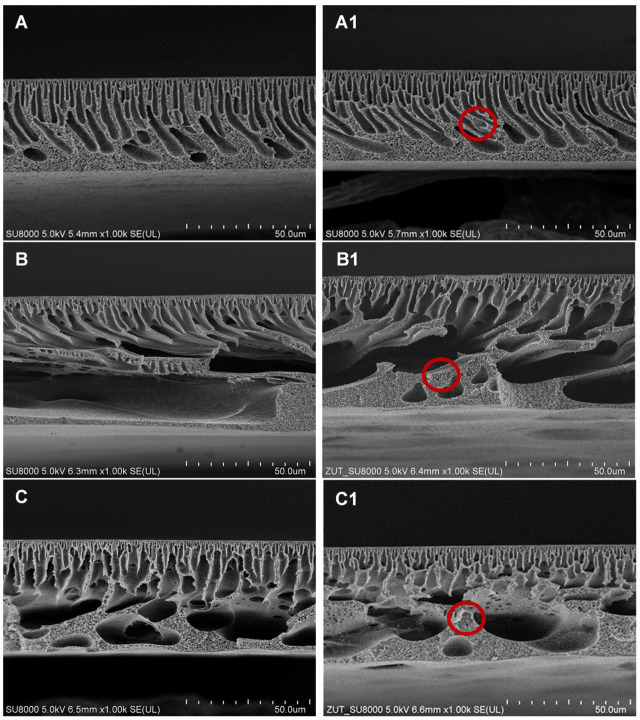
SEM images of the cross-sections of the unmodified (left column, **A**—0%DMF; **B**—0%NMP; **C**—0%DMA) and HNTs-modified membranes (right column, **A1**—0.5%DMF; **B1**—0.5%NMP; **C1**—0.5%DMA).

Figure 1 presents the SEM images of the cross-sections of the membranes prepared using various polymer solvents. All of the membranes exhibit an asymmetric structure with a separation layer on the top. However, the use of different solvents significantly influenced the morphology of the membranes (Figure 1). In the case of the membranes prepared with DMF as a solvent, the most compact structure was obtained. The membranes were characterized by the narrow finger-like pores in the middle part and a spongy structure in the bottom part of the cross-section and between the finger-like pores. The use of both DMA and NMP resulted in the formation of finger-like pores in the upper part and large voids in the bottom part of the membranes. The structure of both types of membranes was more loose compared to that fabricated using DMF. However, similarly to the case of the DMF-based membranes, a spongy structure was observed in the bottom part of the cross-sections.

The differences in the structure of the membranes obtained using various solvents are related to the rate of the solvent and non-solvent exchange (i.e., the liquid–liquid demixing rate). A high miscibility between solvent and non-solvent ensures a rapid exchange of the solvent in the forming membrane with the non-solvent in the coagulation bath [35,39]. As a result of the instantaneous demixing, a fast precipitation of polymer occurs, leading to the formation of a thin skin layer and a relatively loose, porous substructure containing large macrovoids [32,40]. That phenomenon was observed in the case of the membranes fabricated using NMP and DMA (Figure 1B,B1,C,C1). On the contrary, application of DMF resulted in a more compact morphology of the membrane with fewer macrovoids and a sponge-like structure (Figure 1A,A1), which is due to a slower exchange of the solvent with the non-solvent (delayed demixing) [22].

**Figure 2:**
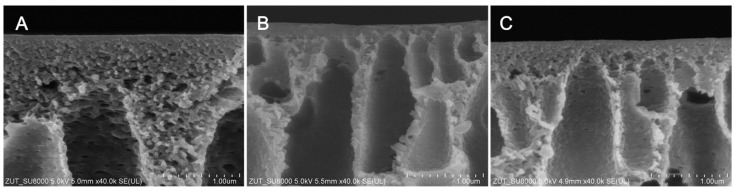
SEM images of the membrane separation layer (**A**—0%DMF; **B**—0%NMP; **C**—0%DMA).

Figure 2 presents SEM images of the separation layer of the membranes prepared using various solvents. The thickness of the skin increased as follows: DMA (~70 nm) < NMP (~75 nm) < DMF (~100 nm). These results are in the agreement with the above-discussed mechanism of demixing. As was already explained, a high miscibility of the solvent with the non-solvent may lead to the formation of a thin separation layer, thereby increasing water permeability [32,40].

In the case of the HNTs-modified membranes, the presence of the nanofiller was confirmed (circles in Figure 1). The distribution of HNTs was random and their presence did not affect the structure or thickness of the membranes. Nonetheless, the membrane thickness was influenced by the type of solvent and increased from ~40 µm for DMF to ~45 µm for DMA and ~55 µm for NMP. The observed increase resulted from a more developed porous structure (especially the presence of macrovoids) for the membranes obtained using DMA and NMP solvents.

**Figure 3:**
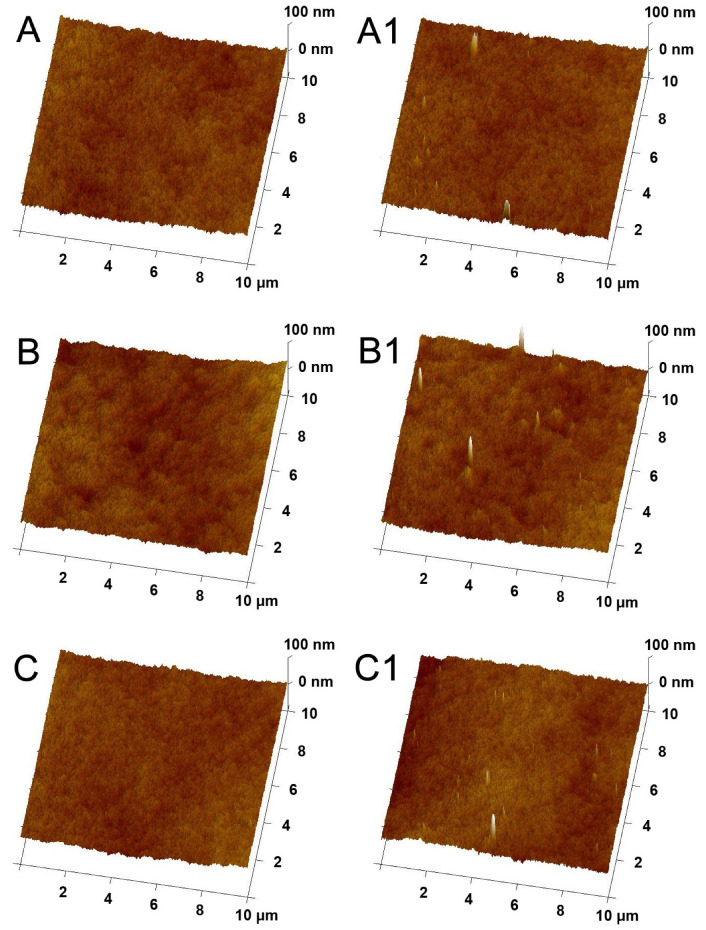
AFM images of the surface of **A**—0%DMF, **A1**—0.5%DMF, **B**—0%NMP, **B1**—0.5%NMP, **C**—0%DMA, and **C1**—0.5%DMA membranes.

Figure 3 shows AFM images of the membranes’ surfaces visualized in 3D mode. In the case of the mixed-matrix membranes, the presence of HNT agglomerates can be observed. On the surface of the membrane prepared with DMF, the agglomerates with a size of ~30 to ~270 nm were found. The surface of the DMA membrane was covered with HNT agglomerates with diameters ranging from ~25 to ~420 nm, but most of them were about 100 nm in size. In the case of the membrane fabricated using NMP, the agglomerates with a diameter in the range of ~25 to 300 nm were present, and most of them exhibited a size of about 80 nm. In the AFM images (Figure 3), the difference in the surface roughness (R_a_) of the obtained membranes was also noticeable.

**Figure 4:**
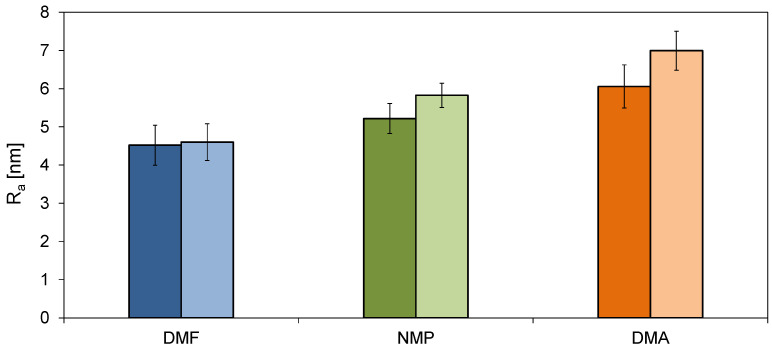
The influence of HNTs and the type of solvent on surface roughness (R_a_) of membranes (for each solvent: the darker, left column corresponds to the unmodified membrane; the lighter, right column represents the modified membrane).

The analysis of the R_a_ value (Figure 4) revealed the highest roughness in the case of the membranes prepared using DMA, while the smoothest surface exhibited the membranes obtained with the application of DMF. The R_a_ value of the unmodified 0%DMF, 0%NMP, and 0%DMA membranes amounted to 4.52(0.52) nm, 5.22(0.39) nm, and 6.06(0.56) nm, respectively. After the introduction of HNTs, the R_a_ increased up to 4.60(0.48) nm, 5.83(0.32) nm, and 6.99(0.51) nm, respectively. The observed changes in roughness are related to the presence of HNTs on the surface of the membranes (Figure 3). A greater increase in R_a_ was noticeable in the case of the modified 0.5%DMA and 0.5%NMP membranes than in the case of 0.5%DMF. In the AFM images of the 0.5%DMA and 0.5%NMP (Figure 3), the presence of a higher number of the NPs agglomerates was found than in the case of 0.5%DMF. The observed differences can be explained in terms of the structure formed during the phase inversion process. The 0%DMF and 0.5%DMF membranes were characterized by a more compact structure than the DMA- and NMP-based membranes (Figure 1). As a result, in the case of the DMF-based membrane, the HNT aggregates were blocked in the sublayer of the membrane at the stage of the solvent–non-solvent exchange. The highest roughness was observed for the DMA-based membranes, which can be attributed to the largest pore diameter from all of the examined membranes (see below). The roughness of the NMP–based membranes was lower compared to the DMA-based membranes, which could be explained by a more compact structure. A similar relationship was observed by Thuyavan et al. [32], who reported an increase in membrane roughness in the following order: DMF < NMP < DMA. A lower surface roughness was explained by a more compact structure and smaller pore diameters of the membranes.

**Figure 5:**
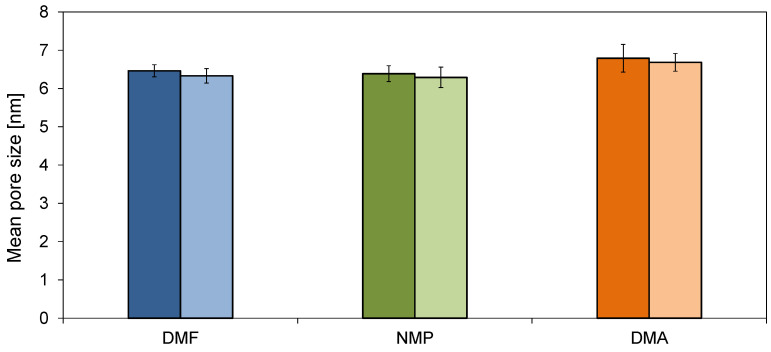
Mean pore size of the neat (the darker, left columns) and modified membranes (the lighter, right columns).

The mean pore size of the obtained membranes is presented in Figure 5. It can be observed that the membranes were characterized by different pore diameters, although the values changed in a relatively narrow range (from 6.3 to 6.8 nm). The largest pore diameters were found in the case of the membranes prepared with the application of DMA as a solvent, and smaller for the NMP- (6.4 nm) and DMF-based (6.5 nm) membranes. The addition of HNTs caused a slight decrease in the pore diameter of all the membranes (to 6.3, 6.3, and 6.7 for the DMF, NMP, and DMA solvents, respectively).

**Figure 6:**
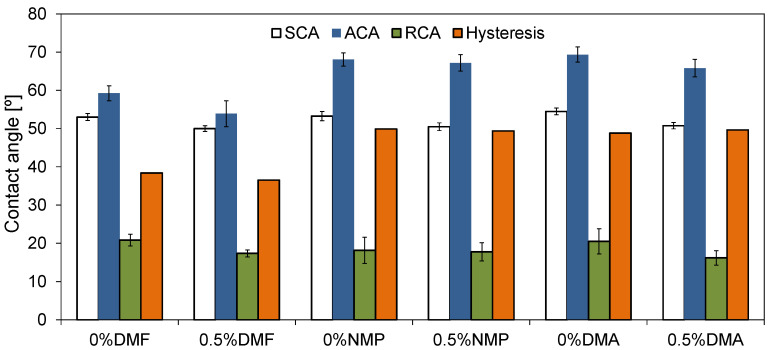
Static, advancing, and receding contact angle of the neat and mixed-matrix membranes.

The membranes obtained with the application of various polymer solvents were characterized by different hydrophilicity. Figure 6 presents static (SCA) and dynamic contact angle values determined for the neat and HNT-modified membranes.

Comparing the effect of the solvent on the SCA of both neat and mixed-matrix membranes, it can be observed that the values follow the order: DMF ≈ NMP < DMA (Figure 6). Moreover, the introduction of hydrophilic HNTs influenced the SCA of all the membranes, regardless of the applied solvent (decrease for 3°). Very different results were obtained by Guan et al. [37] who prepared sulfonated polyethersulfone membranes using the same solvents. The authors observed that the increase in the SCA of the membranes was in the following order: NMP < DMA < DMF. On the contrary, Thuyavan et al. [32] during their investigations on PES (17.5 wt%) membranes observed the lowest hydrophilicity in the case of the application of DMA, and the effect of the solvents on SCA followed the order: DMF < NMP < DMA.

Except for the static contact angles, the values of advancing (ACA) and receding (RCA) contact angles were also determined. ACA is a measure of the overall hydrophobicity of a surface, while RCA is a measure of relative hydrophilicity. Due to their flexibility, polymer surfaces can change their surface energy depending on the surrounding medium. This feature is one of the reasons for the hysteresis observed between ACA and RCA. Moreover, hysteresis depends also on the surface roughness [41,42]. Under certain conditions, the rough surfaces cannot be completely wetted by water and could produce the so-called composite surfaces, in which air becomes trapped in the surface troughs [43,44]. In such a case, the contact angle can be higher than that measured for a smooth surface. On the other hand, when a rough surface is completely wetted (all the cavities/pores are fully filled with water), the contact angle can be lower compared to that measured for a smooth surface [44]. The obtained ACA, RCA, and hysteresis results are shown in Figure 6. In the case of the neat membranes, the lowest ACA was observed for 0%DMF. The value of ACA measured for 0%NMP and 0%DMA was similar to each other and visibly higher compared to 0%DMF. These results suggest that the so-called composite surface mentioned above is formed in the case of the NMP- and DMA-based membranes. The modification with HNTs resulted in a decrease in the value of ACA for all the examined membranes, although the effect was least significant when NMP was used as a solvent. The difference in ACA measured for the neat and mixed matrix membranes reached 5°, 3°, and 1° for the DMF, DMA, and NMP solvents, respectively. The addition of HNTs affected also RCA, which decreased 4° and 5° in the case of DMF and DMA, respectively, while the NMP-based series remained unchanged. The lowering of the contact angle value can be attributed to the presence of -OH groups in the structure of the HNTs [45,46].

Based on the results obtained for the ACA and RCA measurements, the hysteresis was calculated. In the case of the DMF-based membranes, after the introduction of HNTs, a slight decrease in the hysteresis value from 38° to 36° was observed. The use of DMA and NMP as polymer solvents resulted in a higher hysteresis compared to DMF. Moreover, the values were similar for the neat and mixed-matrix membranes and amounted to 49° to 50°. These data show that the effect of modification with the nanofiller was very different for the DMA- and NMP-based membranes compared to the DMF-based one. The decrease of the hysteresis for the 0.5%DMF membrane compared to 0%DMF can be mainly attributed to the lowest roughness of the DMF-based membranes amongst all the examined cases (Figure 4). Similarly, the almost unchanged hysteresis values observed for the neat and mixed-matrix membranes obtained using the two other solvents can be explained by the higher surface roughness of the DMA- and NMP-based membranes compared to the DMF-based series (Figure 4).

### 2.2. Permeability of the Membranes

**Figure 7:**
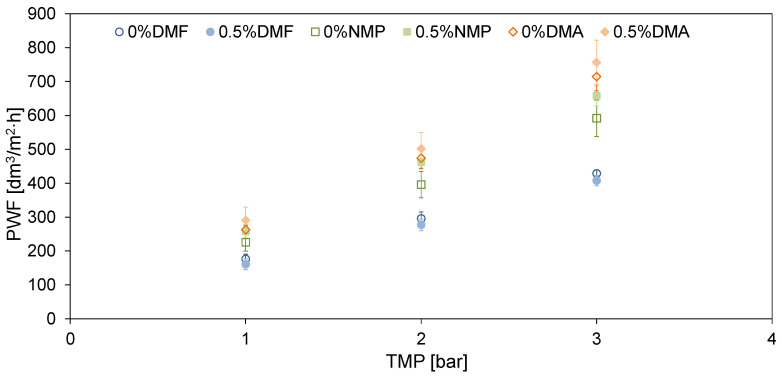
The influence of the addition of HNTs and the type of the solvent on pure water flux through the neat and mixed-matrix PES membranes.

The influence of different solvents on the permeability of the neat and mixed-matrix membranes is summarized in Figure 7.

No improvement of pure water flux (PWF) after the introduction of HNTs to the DMF-based membrane was found. This can be explained by the compact membrane structure (Figure 1) and thick separation layer (Figure 2) which reduced the positive effect of the modification with HNTs. A slight increase in PWF (from 714 to 756 dm^3^/m^2^ h at the transmembrane pressure TMP = 3 bar) was observed after the introduction of HNTs into the matrix of the DMA-based membrane. The most significant effect of the modification with HNTs was found for the NMP-based membrane, in the case of which the PWF increased from 592 to 658 dm^3^/m^2^ h at TMP = 3 bar. The increase in membrane permeability after the incorporation of HNTs can be related to the tubular structure of the nanomaterial and its hydrophilic nature [47]. Despite slight changes in hydrophilicity due to the low nanofiller content, the increase in permeability could be related to the nanofiller distribution in the membrane matrix. The SEM images (Figure 1) show that a significant part of the introduced HNTs formed agglomerates localized in the sublayer of a membrane, which reduced their amount on the membrane surface. The increase in membrane roughness observed for the mixed matrix 0.5%DMA and 0.5%NMP compared to the neat 0%DMA and 0%NMP confirm the presence of a noticeable amount of HNTs on the surface of these membranes. On the contrary, the R_a_ of the 0%DMF and 0.5%DMF membranes was very similar, indicating a low content of the nanofiller on the surface.

Comparing the effect of various solvents on the PWF it can be observed that the flux increased in the order: DMF < NMP < DMA. The differences in permeability can be associated with changes in the structure of the membranes visible in the SEM images (Figure 1 and Figure 2). The use of DMF resulted in the formation of a thicker separation layer, and thus these membranes exhibited lower permeability compared to DMA and NMP-based membranes, with a thinner separation layer [32,40]. A higher permeability of the DMA-based membranes compared to the NMP-based ones can be further explained by a larger pore diameter of the 0%DMA and 0.5%DMA compared to the 0%NMP and 0.5%NMP (Figure 5). A similar relationship between the type of solvent and the PES membranes’ permeability was reported by Thuyavan et al. [32]. The highest value of the flux observed for the membrane prepared with the use of DMA was associated with the formation of larger pores in the structure of the membrane, while the lowest value observed in the case of DMF was attributed to a more compact membrane structure. The obtained results were also explained in terms of a higher viscosity of the solution of polymer in DMF compared to DMA and NMP, which delayed the solvent–non-solvent exchange and thus resulted in a more compact structure characterized by a lower permeability [32].

### 2.3. Separation Properties

**Figure 8:**
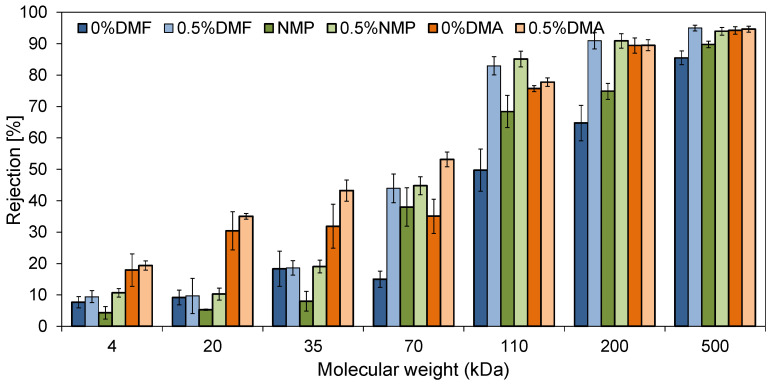
The influence of the addition of HNTs and the type of the solvent on the rejection of PEGs (4 to 35 kDa) and dextrans (70 to 500 kDa) by the neat and mixed-matrix PES membranes.

Figure 8 shows the rejection (R) of model organic compounds, poly(ethylene glycols) (PEGs) and dextrans, by the neat and mixed-matrix membranes prepared using various solvents.

Significant differences in the separation properties were observed between the membranes prepared with different solvents. Comparing the unmodified membranes, the highest PEGs rejection was observed for the 0%DMA membrane (18%, 30%, and 32% for 4, 20, and 35 kDa, respectively). The separation performance of 0%DMF and 0%NMP towards PEGs was visibly lower (18% and 8% for PEG 35 kDa, respectively). In the case of the separation of dextrans, the rejection efficiency followed the order: 0%DMF < 0%NMP < 0%DMA. For the 200 kDa dextran, the R value reached 65%, 75%, and 89%, respectively. Regardless of the polymer solvent applied, the introduction of HNTs to the membranes’ structure resulted in an improvement in the separation properties. The rejection of 35 kDa PEG by 0.5%DMF, 0.5%NMP, and 0.5%DMA reached 19%, 19%, and 43%, respectively, whereas the rejection of 200 kDa dextran amounted to 91%, 91%, and 90%, respectively. The separation mechanism of ultrafiltration is based on a sieve effect and depends mainly on the size and shape of the pores on the membrane surface. The observed improvement in the separation performance of the membranes after the introduction of the nanofiller can be attributed to the additional pores of HNTs (lumen and surface pores and defects) and voids formed between the HNTs tubes forming agglomerates. Furthermore, the enhancement of the separation efficiency was explained by a tortuous path of water flowing through the membrane caused by the agglomerates as well as by the unique adsorption property of HNTs due to their positively charged inner and negatively charged outer surfaces [10]. Nonetheless, no direct correlation between the pore size determined on the basis of the AFM analysis (Figure 5) and the rejection of PEGs and dextrans (Figure 8) was found. The 0%DMA membrane characterized by the largest mean pore size exhibited the highest rejection of the model compounds. However, the 0%DMF membrane, possessing a similar pore size to 0%NMP, exhibited a lower dextrans separation efficiency than the 0%NMP membrane, while after the introduction of HNTs the performance of both types of membranes was similar. In contrast to flow-through measurement techniques, the AFM microscopy does not allow for the differentiation between open and closed pores in the membrane separation layer. It is possible that the 0%DMF membrane had more of the open pores, which allowed the model compounds to pass through, compared to the larger, but likely closed pores in the 0%DMA membrane. Also, this technique does not allow for the measurement of the pores within the HNTs, whose presence also contributes to the rejection of the model compounds. These data show that the evaluation of the separation performance of the membranes using model solutes is a more appropriate approach than the determination of the mean pore size on a basis of the AFM analysis.

### 2.4. Antifouling Properties

**Figure 9:**
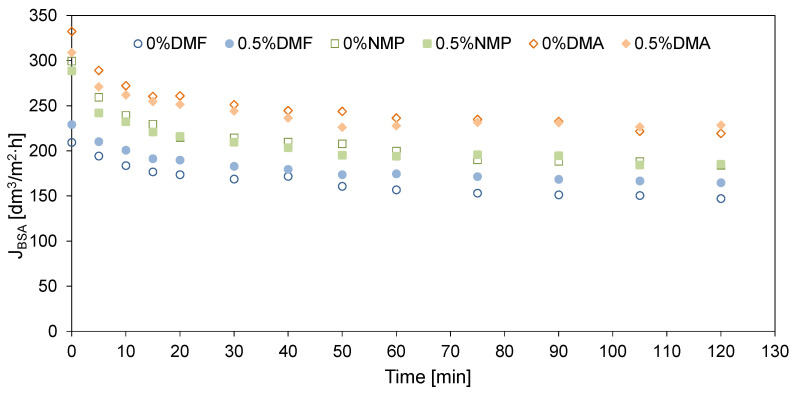
The effect of the type of solvent and the addition of HNTs on BSA fouling of the PES membranes. Initial BSA concentration: 1 g/dm^3^; TMP = 2 bar.

The antifouling properties of the membranes were determined using bovine serum albumin as a model foulant (Figure 9). The ultrafiltration process was carried out for 2 h, during which a decrease in the permeate flux was observed over time. The use of HNTs had a noticeably positive effect on the fouling mitigation only in the case of the DMF-based membranes. The permeate flux at the end of the process reached 147 dm^3^/m^2^ h for 0%DMF and 165 dm^3^/m^2^ h for 0.5%DMF. In the case of the NMP- and DMA-based membranes, no significant effect of the HNTs addition on the membrane fouling alleviation was observed. At the end of the experiments, the flux reached 184 and 185 dm^3^/m^2^ h for 0%NMP and 0.5%NMP, and 220 and 229 dm^3^/m^2^ h for 0%DMA and 0.5%DMA, respectively. The higher values of the permeate flux observed for the DMA and NMP-based membranes compared to the DMF-based ones resulted from the higher water permeability of the former membranes (Figure 7).

The decrease in the permeate flux through the unmodified 0%DMF membrane in comparison to PWF reached 54% after 2 h of the experiment, whereas for the 0.5%DMF, the permeate flux was reduced by 40%. In the case of the neat 0%DMA and 0%NMP, the decrease in permeate flux was 53%. After modification, there was a 53% decrease of 0.5%DMA and 55% for 0.5%NMP. The improved fouling resistance in the case of 0.5%DMF compared to 0%DMF can be related to the lower roughness of the DMF-based membranes compared to the NMP- and DMA-based membranes. Another explanation can be its low permeability. Conversely, the increase in roughness and PWF value upon modification with HNTs resulted in almost unchanged fouling resistance of the 0.5%NMP and 0.5%DMA membranes compared to the neat 0%NMP and 0%DMA. Thuyavan et al. [32] also related the fouling susceptibility of the membranes prepared with various solvents to the differences in the surface roughness. The authors reported that the membrane prepared with the use of DMF had the highest fouling resistance, which was ascribed to its low roughness, high hydrophilicity, and small pore diameter. The fouling resistance was also influenced by the hydrophilicity of the tested membranes (Figure 6). An increase in the hydrophilicity of a membrane results in a reduction in the hydrophobic interactions between the membrane surface and the BSA molecules, which can lead to the adsorption of the foulant on the surface or the formation of a permeation-limiting hydrophobic layer [48,49]. Although the SCA values of all the examined membranes were comparable (50° to 54°), a significant difference was found in the case of ACA (Figure 6). As was already explained, ACA represents the overall hydrophobicity of the membrane surface. The value of ACA of the NMP- and DMA-based membranes was visibly higher (66° to 69°) compared to that of the DMF-based membranes (54° to 59°), indicating a higher hydrophobicity of the surface of the former membranes compared to the latter ones.

### 2.5. Antibacterial Properties

**Figure 10:**
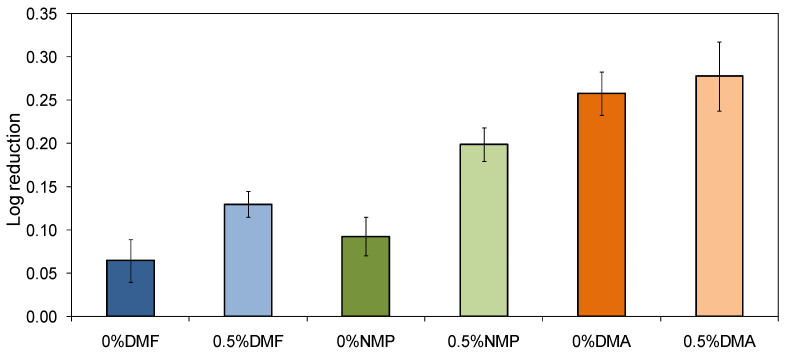
Inhibition of *E. coli* growth in the presence of the neat and mixed-matrix membranes.

The antibacterial properties of the neat and mixed-matrix membranes are shown in Figure 10.

It was found that both the type of the solvent and the addition of HNTs had an influence on the inhibition of *E. coli* growth in the presence of the examined membranes. The highest antibacterial activity was observed for the DMA-based membranes, while the lowest was observed for the DMF-based series. Moreover, the incorporation of HNTs into the membranes’ matrix improved the antibacterial properties of the membranes, regardless of the applied solvent. In the case of the 0%DMF membrane, the number of bacterial colonies decreased by only 14%, while after the modification with HNTs the percentage reduction was almost twice as high and reached 26%. A similar improvement of the antibacterial properties was observed for the 0.5%NMP membrane, for which the percentage reduction was 37%. In the case of the neat 0%NMP, it was 19%. The influence of HNTs on the antibacterial performance of the DMA-based membranes was less pronounced. The number of bacterial colonies decreased by 45% in the presence of the neat 0%DMA and for 47% in the case of the mixed-matrix 0.5%DMA.

**Figure 11:**
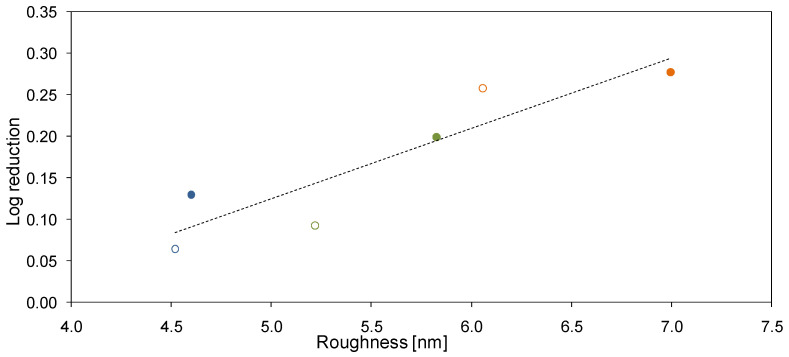
Influence of roughness on antibacterial activity of the neat (open circles) and mixed matrix (closed circles) PES membranes.

The antibacterial properties of the neat and mixed matrix membranes can be explained in terms of mechanical damage to bacterial cells. Such damage might be induced by a rough surface of the membranes. Figure 11 confirms that a certain correlation between the R_a_ and antibacterial activity of the obtained membranes exists. Furthermore, it can be found that the antimicrobial properties of the membranes can be changed by changing the polymer solvent even in the absence of the HNTs. Nonetheless, the application of HNTs contributes to the enhancement of the antibacterial properties of the membranes. A similar phenomenon was observed during our previous studies on PES membranes modified with titanate nanotubes [50]. HNTs, likewise TNTs, exhibit a needle-like structure that can penetrate the outer layer of the *E. coli* cells leading to their damage. This type of mechanism of bacterial inactivation has also been observed in the case of membranes modified with CNTs [51,52].

## 3. Materials and Methods

Polyethersulfone (Ultrason E6020P) was provided by BASF SE (Ludwigshafen, Germany). DMF (puriss p.a.) was purchased from Avantor Performance Materials Poland S.A. (Gliwice, Poland), while DMA and NMP were obtained from Acros Organics (Geel, Belgium and the Netherlands, respectively). Halloysite nanotubes were supplied by Sigma Aldrich (Saint Louis, MO, USA). The length of HNTs ranged from 150 to 1250 nm, the inner diameter was in the range of 11 to 28 nm and the wall thickness was 5–23 nm. The detailed characteristics of HNTs are presented elsewhere [53]. Bovine serum albumin (Probumin) was purchased from Merck (Darmstadt, Germany). Poly(ethylene glycols) were provided by Sigma Aldrich (Steinheim, Germany) and dextrans by Polfa Kutno (Kutno, Poland). In all experiments, pure (deionized) water (type 2, 0.066 μS/cm) from Elix 3 (Millipore, Burlingtone, MA, USA) was applied.

Plate count agar (BIOCORP, Warszawa, Poland) and NaCl (Merck, Søborg, Denmark) were used in microbiological tests. The initial concentration of the Gram-negative *Escherichia coli* (ATCC 29425, Manassas, VA, USA) suspension was set at 0.5 according to the McFarland scale (McFarland standards, bioMérieux, Marcy l’Etoile, France).

PES UF membranes were prepared by the wet phase inversion method. In the case of the unmodified membrane, the polymer (15 wt%) was dissolved in DMF, DMA, or NMP (85 wt%), respectively. The homogeneous casting dope was cast onto a glass plate using an automatic film applicator (Elcometer 4340, Elcometer Ltd., Manchester, UK) with the knife gap of 0.1 mm, and subsequently immersed in a pure water bath (type 2, 0.066 μS/cm, Millipore, Burlingtone, MA, USA, 20 ± 1 °C) to complete the phase inversion process.

The casting dope for the fabrication of the mixed-matrix membranes was obtained by mixing a dispersion of HNTs in an appropriate solvent: DMF, DMA, or NMP (10 cm^3^) with the previously prepared solution of PES in the solvent (40 cm^3^). The dispersion of HNTs in a solvent was prepared by sonication for 30 min using an ultrasonic probe (Vibra-cell VCX-130, Sonics, Randor, PA, USA; output power 130 W, frequency 20 kHz, amplitude 80%). After addition of the HNTs and their dispersion into the polymer solution, the casting dope was mixed alternately using: (i) a magnetic stirrer at the temperature of 55 to 60 °C and (ii) sonication in an ultrasonic bath (Sonic-6D, Polsonic, Poland; output power 320 W, frequency 40 kHz) for 2 h, 15 min by turns. After degassing of the casting dope at room temperature, the membranes were cast using the automatic film applicator as described above. The content of HNTs in the casting dope was 0.5 wt% (by weight of the polymer). The amount of HNTs was selected on the basis of the previous work [53], which revealed that the membrane containing 0.5 wt% of HNTs showed the best fouling resistance, good dispersion of HNTs on the surface, and relatively low roughness.

The static contact angle of the membranes was determined by a sessile drop approach using a goniometer (type 260 ramé-hart instruments co., Succasunna, NJ, USA). The applied volume of the water drop was 10 µL. The results are mean values of at least 10 measurements. Moreover, the advancing and receding contact angles were determined by slowly increasing (from 3 to 11 µL) and subsequently reducing the volume of water drop. The results are mean values of at least five measurements.

The surface topography of the membranes was analyzed on a basis of AFM images collected using an atomic force microscope (NanoScope V Multimode 8, Bruker Corp., Billerica, MA, USA) in the ScanAsyst mode. The silicon nitride probe was applied. The average roughness value (R_a_) of the membrane surface was determined in the NanoScope Analysis software using at least five AFM images (10 × 10 µm). The size of the membrane pores in the skin layer was calculated based on the AFM images (1 × 1 μm) with the application of the Gwyddion software package using a slightly modified methodology described by Khanukaeva et al. [54]. The images were first flattened to the third order using the NanoScope Analysis software. In order to define the pore diameters, both the threshold and the watershed modes were applied. In the threshold mode, the AFM image is cut horizontally at a certain height (the threshold). The resulting plane contains some irregular shaped areas, which are considered as pore openings. For the proper identification of the pores by this method, the analyzed surface must be flat enough, i.e., free of deformations. However, even with the flattening procedure the shapes that could be considered to be the pores were not completely identified by the threshold approach. Therefore, the watershed method was also used. In this method, the analyzed surface is covered with the virtual water droplets and then a numerical simulation of their free spreading is conducted. The surface porosity is analyzed based on the position, size, and shape of such created structures which are interpreted as pores. A more detailed description of the method can be found elsewhere [54].

The morphologies of the membranes’ cross-sections were examined using the ultra-high-resolution field-emission scanning electron microscope (UHR FE-SEM) Hitachi SU8020 (Düsseldorf, Germany). The samples of membranes were dehydrated in ethanol and then broken in liquid nitrogen. Before the measurement, the samples were sputtered with a chromium layer (Q150T ES Quorum Technologies Ltd., Lewes, UK). The analysis was carried out using the secondary electrons (SE) mode (accelerating voltage 5 kV).

The antibacterial properties of the membranes were evaluated using *Escherichia coli* as a model microorganism. A piece of a membrane (12.5 × 4.5 cm) was dehydrated in ethanol, dried in air, and put into a glass bottle filled with 100 cm^3^ of *E. coli* suspension. A control sample was prepared in the same way but without a membrane. Three separate experiments were realized to confirm the reproducibility of the results. The samples were incubated at 37 °C for 24 h under a continuous stirring using a magnetic stirrer (250 rpm). The number of bacteria was determined using the decimal dilutions in a sterile NaCl (8.5 g/dm^3^) solution. A total of 0.3 cm^3^ of the diluted solution was spread using a spreader on the PCA plate. The agar plates were incubated at 37 °C for 24 h. The visible colonies were counted by the counter (LKB 2002, POL-EKO, Wodzisław Śląski, Poland). The results were calculated as the average colony-forming units (CFU) according to Equation (1):

CFU/cm^3^ = (N × Y)/Z (1)

where N denotes the number of bacteria colonies visible on agar plates, Y denotes the total dilution, and Z denotes the volume of bacteria suspension (0.3 cm^3^).

The log reduction of bacterial growth was determined with reference to the control sample using Equation (2):

Log reduction = log (A/B)(2)

where A denotes the number of bacteria in the control sample and B denotes the number of bacteria in the presence of a membrane.

The pure water flux, as well as the separation and antifouling properties, were examined based on a cross-flow UF process with the application of an experimental setup described elsewhere [55]. The experiments were repeated at least three times to confirm the reproducibility of the results. The membrane (0.0025 m^2^) was mounted in a stainless-steel module with a 1.194 mm feed spacer. The PWF was evaluated by ultrafiltration of pure water (type 2, 0.066 μS/cm, Millipore, 20 °C) at the transmembrane pressures (TMPs) 1, 2, and 3 bar. Antifouling properties of the membranes were determined on a basis of 2 h of ultrafiltration of BSA solution (1 g/dm^3^). The feed cross-flow velocity and TMP during the fouling experiments were 1 m/s and 2 bar, respectively.

Separation properties of the membranes were evaluated during the ultrafiltration of a series of model solutions containing poly(ethylene glycols) (PEGs) with molecular weights of 4, 20, and 35 kDa, and dextrans with molecular weights of 70, 110, 200, and 500 kDa. The concentration of the model organic compounds was 0.5 g/dm^3^, and the TMP was set at 1 bar. The rejection of PEGs and dextrans was calculated based on Equation (3):

R = [(C_0_ − C_p_)/C_0_] × 100% (3)

where C_0_ is the concentration of PEG and dextrans in the feed, while C_p_ is the concentration in permeate.

The concentration of the model compounds in the feed and permeate was measured using high-performance liquid chromatograph LaChrom Elite (Hitachi, Chiyoda, Tokio, Japan) equipped with the refractive index detector L-2490 and the PolySep-GFC-P4000 column (Phenomenex, Torrance, CA, USA). Ultrapure water was used as a mobile phase.

## 4. Conclusions

The influence of the polymer solvent on the physicochemical, transport, separation, antifouling, and antibacterial properties of PES mixed matrix membranes modified with HNTs was presented and discussed. Membranes prepared with DMF were characterized by a dense structure with a thick separation layer and some finger-like pores in the sub-layer, while in the cross-section of DMA- and NMP-based membranes, a thin skin in the top and macrovoids in the bottom part were noticed. These differences in the morphology were related to the various mechanisms of phase inversion during membranes’ formation, i.e., delayed demixing in the case of DMF and instantaneous demixing in the case of DMA and NMP solvents. AFM analysis revealed the presence of the HNT aggregates on the surface, which influenced the roughness of the obtained membranes. The highest R_a_ value was observed for the DMA-based membranes, while the lowest was observed for the DMF-based ones. The static contact angles changed in the following order: DMF ≈ NMP < DMA indicating similar hydrophilicity of the DMF- and NMP-based membranes. However, the values of ACA, which can be regarded as a measure of membranes’ hydrophobicity, were visibly higher in the case of the membranes fabricated using NMP and DMA compared to that obtained with the application of DMF.

A higher water permeability was found in the case of the membranes prepared using DMA and NMP as solvents compared to the DMF-based membranes. Moreover, an increase of PWF upon addition of HNTs to the NMP- and DMA-based membranes was observed, whereas the introduction of the nanofiller did not affect the permeability of the membranes obtained using DMF. These results were related to the distribution of the nanomaterial in the matrix and on the surface of the membranes, and the thickness of the separation layer.

The rejection of PEGs and dextrans by the 0%DMF and 0%NMP was visibly lower compared to that by 0%DMA. The introduction of HNTs to the membrane matrix had a positive effect on the separation performance of the membranes, regardless of the applied solvent. No correlation between the pore size determined by AFM and the rejection of the model compounds was found.

A positive influence of the introduction of HNTs to the membrane matrix on the antifouling properties was observed only for the DMF-based membranes. In the case of the membranes prepared using the other solvents the permeate fluxes before and after modification with HNTs were similar. The different effect of the modification was explained in terms of higher permeability of the NMP- and DMA-based membranes compared to the DMF-based ones, as well as their higher overall hydrophobicity represented by the ACA values.

A relationship between the antimicrobial properties and the surface roughness of the membranes was observed. The membranes prepared with the use of DMA as a solvent were characterized by the highest roughness and also by the most efficient inhibition of the *E. coli* growth. The incorporation of HNTs increased the membrane roughness thus further improving the antibacterial performance of the membranes. The results were explained by the mechanical damage of bacterial cells in contact with the rough surface, which limits their growth.

## Data Availability

The data presented in this study are available on request from the corresponding author.

## Sample Availability

Samples are not available.

## Abbreviations and Symbols

A	Number of bacteria in the control sample
ACA	Advancing contact angle
AFM	Atomic force microscopy
B	Number of bacteria in the presence of a membrane
BSA	Bovine serum albumin
C_0_	Concentration of PEG and dextrans in feed
C_P_	Concentration of PEG and dextrans in permeate
CA	Contact angle
CFU	Colony forming unit
CNTs	Carbon nanotubes
DMA	*N,N*-dimethylacetamide
DMF	*N,N*-dimethylformamide
DMSO	Dimethyl sulfoxide
HNTs	Halloysite nanotubes
N	Number of bacteria colonies visible on agar plates
NIPS	Non-solvent-induced phase separation
NMP	1-methyl-2-pyrrolidinone
NPs	Nanoparticles
PEGs	Poly(ethylene glycols)
PES	Polyethersulfone
PLA	Poly(lactic acid)
PWF	Pure water flux
R	Rejection
R_a_	Mean roughness (roughness average)
RCA	Receding contact angle
SCA	Static contact angle
SE	Secondary electrons
SEM	Scanning electron microscopy
TMP	Transmembrane pressure
TNTs	Titanate nanotubes
UF	Ultrafiltration
Y	Total dilution
Z	Volume of bacteria suspension
